# Genistein exerts anti-colorectal cancer actions: clinical reports, computational and validated findings

**DOI:** 10.18632/aging.204702

**Published:** 2023-05-07

**Authors:** Xiaoxia Liu, Ying Lan, Li Zhang, Xi Ye, Qingrong Shen, Guangyan Mo, Xiaoyu Chen

**Affiliations:** 1 Department of Pharmacy, Guangxi Academy of Medical Sciences and the People’s Hospital of Guangxi Zhuang Autonomous Region, Nanning 530021, Guangxi, People’s Republic of China

**Keywords:** clinical properties, colorectal cancer, genistein, bioinformatics, autophagy

## Abstract

Colorectal cancer (CRC) is presently a health challenge in China. Although clinical chemotherapy is prescribed availably, the negative effects and poor prognoses still occur. Genistein has antitumor properties in our previous studies. However, the molecular mechanisms underlying the anti-CRC effects of genistein remain unclear. Increasing evidences have indicated that the induction of autophagy, one of cell death models, is closely associated with the formation and development of human cancer. In the current study, a systematic bioinformatics approach using network pharmacology and molecular docking imitation was aimed at identifying the pharmacological targets and anti-CRC mechanisms of genistein, characterized by autophagy-related processes and pathways. Moreover, experimental validation was conducted by using clinical and cell culture samples. All 48 potential targets of genistein-anti-CRC-associated autophagy were screened accordingly. Further bioinformatics analyses identified 10 core genistein-anti-CRC targets related to autophagy, and enrichment-assayed results revealed that the biological processes of these core targets might regulate multiple molecular pathways, including the estrogen signaling pathway. Additionally, molecular docking data demonstrated that genistein has a high affinity for epidermal growth factor receptor (EGFR) and estrogen receptor 1 (ESR1). Both EGFR and ESR1 proteins were highly expressed in clinical CRC samples. Preliminary *in vitro* data showed that genistein effectively reduced cellular proliferation, activated apoptosis, and suppressed EGFR and ESR1 protein expressions in CRC cells. Our research findings uncovered the molecular mechanisms of genistein against CRC, and the potential drug targets associated with autophagy in genistein treatment of CRC were identified and validated experimentally, including EGFR and ESR1.

## INTRODUCTION

Colorectal cancer (CRC) is the third most leading cause of cancer death in the world, accompanied by invasive and metastatic outcomes [1]. CRC is the third most clinically diagnosed cancer in China, characterized with increased deaths and economic burden [2]. It is reported that elevated incidence and mortality of CRC in China are involved in high red and processed meat consumption, low vegetable and fruit uptake, excessive alcohol drinking and tobacco smoking, physical inactivity [3]. Although modern medical screening has progressed, most CRC cases are diagnosed as middle- or late-stage at the first examination [4]. Endoluminal surgery and local excision can be used in clinical CRC management, however, these techniques are still considered to be challenging over time [5]. In the routine clinical management of CRC, existing pharmacological treatments are still associated with unavoidable adverse actions, drug tolerance, and poor prognosis after long-time use [6]. Therefore, candidate adjuvant or alternative treatments characterized by potent efficacy and low toxicity are warranted to enhance the quality life of patients with CRC and extend their survival rate. Autophagy is a pathophysiological condition in the cellular or organelle degradation process that is involved in cell function and fate [7]. Other studies have indicated that autophagic dysfunction is closely associated with human tumorigenesis through affecting neoplastic development, immunity and treatment [8]. In the development of CRC, autophagy-regulated genes and proteins, including catenin beta 1, lysosomal associated membrane protein 1, microtubule associated protein 1 light chain 3, correlate with the initiation and progression of CRC [9]. In CRC, the dual effects of autophagy on tumor promotion and inhibition are widely recognized. Therefore, clarifying the biological effect of autophagy on CRC cells may provide a more substantial theoretical basis for effective CRC treatment [10]. The combined use of autophagic inhibitors and chemotherapy may be a promising treatment for human CRC by regulating the IL-6/JAK2/BECN1 signaling pathway [11]. Other preclinical studies have shown that modulation of the autophagic pathway for inducing cell death may sensitize chemotherapy effectiveness against CRC [12]. Therefore, targeted treatment utilizing autophagy may be promising for patients with CRC. Traditional Chinese medicine (TCM)-derived extracts or compounds are commonly used for potentials in prophylaxis and treatment of human diseases, including cancers and neurodegenerative disorders. And autophagy, a potential drug target, may be mediated functionally by bioactive compounds isolated from TCM [13]. Genistein, a chemical structure known as 4’,5,7-trihydroxyisoflavone, is a natural isoflavone with potent health benefits, including chemoprevention against human cancers and cardiovascular protection [14, 15]. Many experimental studies have demonstrated that genistein exerts anti-CRC action through different signaling pathways, such as Notch1/NF-κB/slug/E-cadherin pathway, ATM/p53 molecular pathway [16, 17]. Our previous studies have suggested that genistein has anti-invasive and anti-proliferative effects on human CRC cells through inhibiting cell proliferation, inducing apoptosis [18, 19]. An *in vitro* study indicated that the combined use of genistein and indol-3-carbinol may induce apoptosis in CRC cells by activating autophagy [20]. Although underlying mechanisms in genistein against CRC is reported, more complete anti-CRC mechanisms targeting autophagy need to be explored. Preclinical bioinformatics has advanced significantly, and the application of network pharmacology has recently been developed to reveal the complete target and mechanism of natural compounds against cancers [21]. The network pharmacology approach, accompanied by molecular docking imitation, has been used in pharmacology research and development for potential drug against cancers [22–23]. Therefore, our study aimed to use comprehensive bioinformatics approach, including network pharmacology and molecular docking, to reveal the anti-CRC biotargets and molecular mechanisms of genistein-induced autophagy. Furthermore, the bioinformatic findings were validated experimentally by using human and cell culture samples. Taken together, the current findings may provide insights to identify that genistein may be a promising medication for treating patients with CRC in future clinical application.

## MATERIALS AND METHODS

### Databases used for screening targets in genistein, autophagy, and CRC

The keyword of “genistein” was used to screen the target genes using Traditional Chinese Medicine Database and Analysis Platform (TCMSP) [24], Bioinformatics Analysis Tool for Molecular mechANism of TCM (BATMAN-TCM) [25], SuperPred [26], and SwissTargetPrediction [27] databases. Canonical targets of autophagy were obtained from GeneCards [28], National Center for Biotechnology Information (NCBI), and Online Mendelian Inheritance in Man (OMIM) [29] databases. Finally, the GeneCards, OMIM, and DrugBank [30] databases were used to screen CRC targets. All unmet targets were rectified through the Uniprot [31] database reviewed (Swiss-Prot) and Human.

### Acquisition of intersection targets in autophagy-genistein-CRC

The intersection targets within autophagy, genistein and CRC were screened and mapped using Venn Diagrams software, as described previously [32].

### Network construction and core target identification

The STRING database [33] was used to construct a protein-protein interaction (PPI) network for genistein-anti-CRC interactions to determine the functional interactions among target protein nodes. The parametrical confidence score of 0.09 was used to obtain candidate targets with “Homo sapiens” that were identified in the Cytoscape software [34]. The Cytoscape-based NetworkAnalyzer plug-in was used to produce the core targets in genistein-anti-CRC, determined from the screening degree values.

### Enrichment analysis of core target genes

R software with the GOplot package and Database for Annotation, Visualization and Integrated Discovery (DAVID) was used to analyze the Gene Ontology (GO) and Kyoto Encyclopedia of Genes and Genomes (KEGG) pathway to reveal the key biological functions and molecular pathways targeting autophagy-associated genes in genistein against CRC. Genistein exerted potential anti-CRC effects by modulating the biological functions of the target genes and pharmacological pathways. Bubble graphs were plotted to visualize the enrichment analysis results. The protocols used were obtained from a published study [35].

### Molecular docking procedures

A molecular docking evaluation was conducted on core target proteins with larger scores using the interactive network genistein-anti-CRC core target data. The chemical and crystal structure files were obtained from the NCBI PubChem (https://pubchem.ncbi.nlm.nih.gov/) and the Protein Data Bank (https://www.rcsb.org/) databases. The binding energy of these structures were optimized using ChemBio3D Draw in ChemDraw software [36]. The target proteins/crystals were then hydrogenated and charges balanced using AutoDock Vina [37]. Then, the selected data were converted to the pdbqt format. AutoDockTools were used to assess the affinity between genistein and the protein models. The molecular docking complexes of the ligand and binding residues were visualized using PyMOL software [38].

### Human CRC sampling

The clinical design using human CRC samples was implemented in accordance with the Code of Ethics of the World Medical Association (Declaration of Helsinki). All five patients with CRC were recruited at the Department of Oncology in People’s Hospital of Guangxi Zhuang Autonomous Region before being diagnosed using medical imaging and pathological tests. Human CRC samples were prepared as 5-μm paraffin-embedded sections for hematoxylin-eosin (HE) stain and immunofluorescence analysis.

### Cell culture and biochemical analysis

A human CRC cell line, named HCT116, was used to assess the pharmacological effectivity of genistein against CRC. The cells were cultured in Dulbecco’s modified Eagle’s medium (DMEM; Solarbio, Beijing, China) supplemented with 10% fetal bovine serum (Dibo Biotechnology, Shanghai, China) and 1% antibiotics (Solarbio). The cells were then placed in an incubator at 37° C and 5% CO_2_. Cells were treated with genistein as they grew at 0, 25 and 50 μM doses for 48 h. The cells were then collected for further cell proliferation assessment and immunofluorescence testing. Cell counting kit-8 (CCK-8) was applied for cell proliferation test in genistein-treated cells, and immunofluorescent staining procedure for apoptosis, EGFR ESR1, was reported previously [39].

### Statistical analysis

Statistical Product and Service Solutions (SPSS, Chicago, IL, USA) software was used for the statistical analysis. All data are expressed as mean ± standard deviation (SD). One-way analysis of variance was used to compare the differences between different comparisons. Statistical significance was set at *p* < 0.05.

## RESULTS

### Identification of autophagy-genistein-CRC targets and construction of correlative network

**Figure 1:**
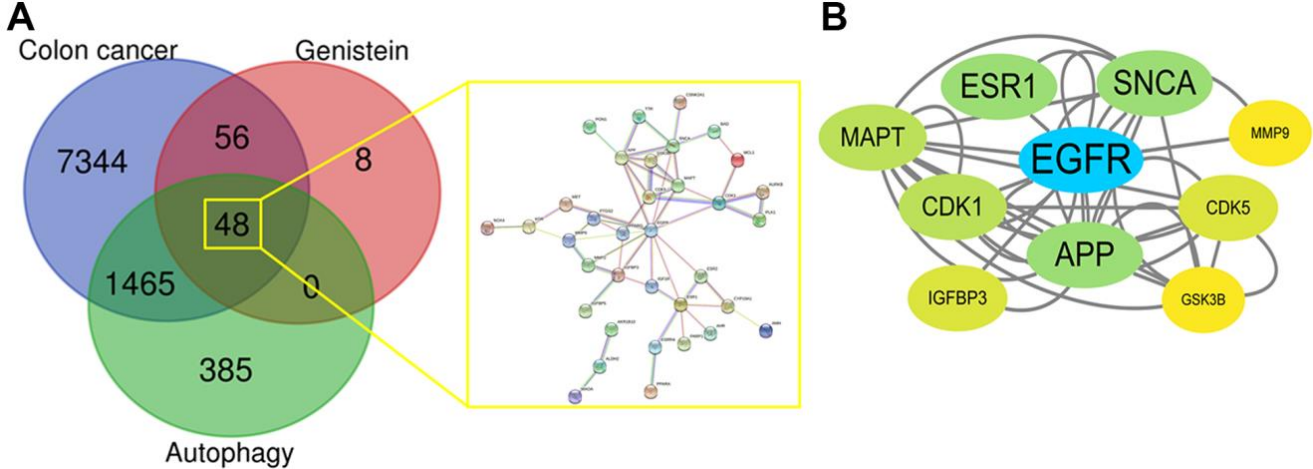
(**A**) Venn diagrams aimed to show respective targets in genistein, CRC, and autophagy before identifying mutual targets within genistein-CRC-autophagy. And all mutual targets were highlighted in the gene-connected network. (**B**) All core target genes in genistein-CRC relating autophagy were identified through highest screening scores.

A total of 1898 autophagy, 112 genistein, and 8913 CRC targets were identified. As a result, 48 mutual targets within autophagy-genistein-CRC were obtained using the Venny tool in Venn Diagrams (Figure 1A). These target genes were used for further analysis using the STRING database for construction network. The PPI network diagram is presented in Figure 1A.

### Identification of the core targets

Cytoscape 3.7.1 software was used to determine the parametric scores, and the screening range for core targets was set to 4–24. A computational assay was conducted for individual target genes and all candidate genes with significant scores were collected accordingly. Finally, 10 core targets with the highest connection degree were identified for genistein against CRC relating autophagy, these included MAPT, ESR1, SNCA, MMP9, EGFR, CDK1, APP, CDK5, IGFBP3, and GSK3B (Figure 1B).

### Functional findings in enrichment analysis

**Figure 2:**
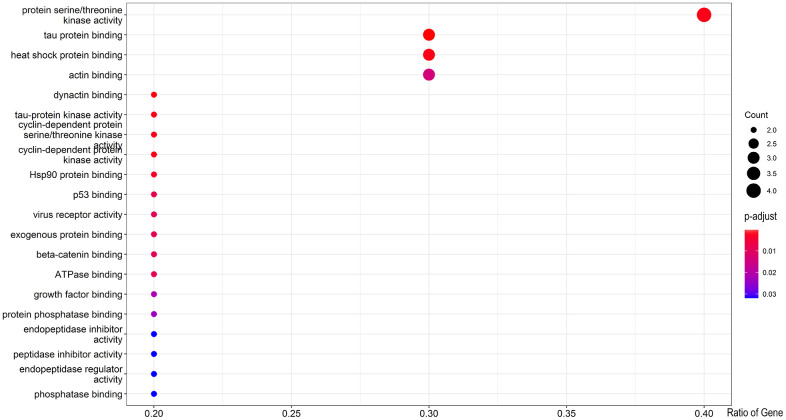
All core targets were used for GO functional enrichment analysis, and biological processes of genistein anti-cancer activity against CRC were revealed in bubble graphs.

**Figure 3:**
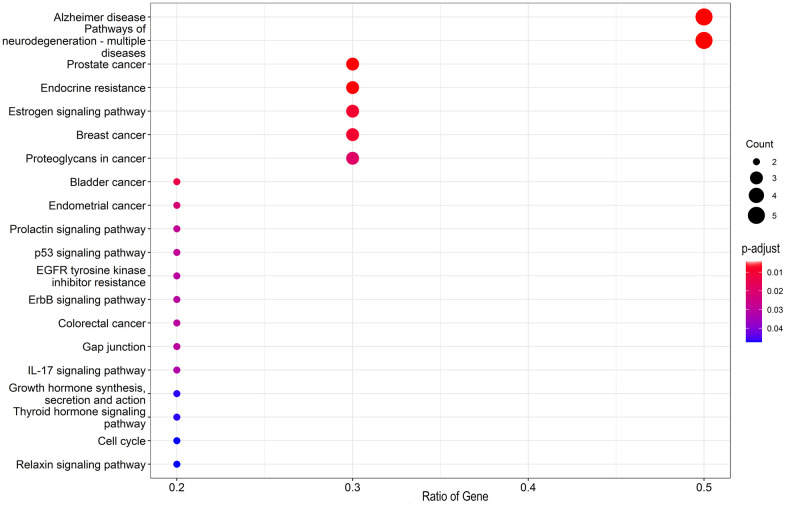
The core targets were used for KEGG pathway enrichment analysis, and molecular mechanisms underlying genistein activity against CRC were uncovered in bubble graphs.

**Figure 4:**
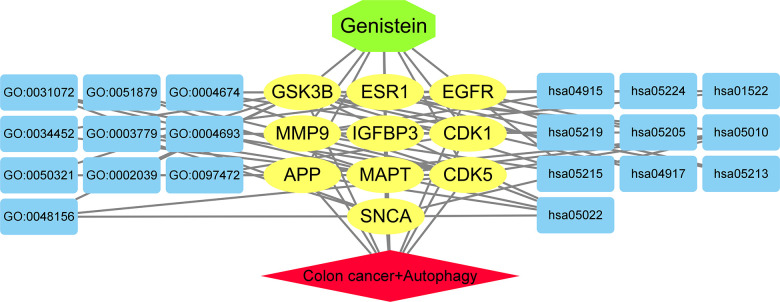
The association network among target genes and pathways associated with autophagy in genistein against CRC were highlighted connectedly.

The GO-related annotation results from core target genes revealed that genistein against CRC targeting autophagy proteins were involved in the regulation of biological processes, including tau protein binding, dynactin binding, heat shock protein binding, protein serine/threonine kinase activity, tau-protein kinase activity, cyclin-dependent protein serine/threonine kinase activity, and Hsp90 protein binding (Figure 2). KEGG enrichment data indicated that these core target genes in genistein against CRC were intimately associated with Alzheimer’s disease, pathways of neurodegeneration-multiple diseases, prostate cancer, endocrine resistance, estrogen signaling pathway, and breast cancer (Figure 3). Molecular pharmacotherapy commonly functions at different cellular and molecular levels, as demonstrated by multiple targets and pathways in genistein against CRC targeting autophagy. The visualization network results were shown in Figure 4.

### Molecular docking validation

**Figure 5:**
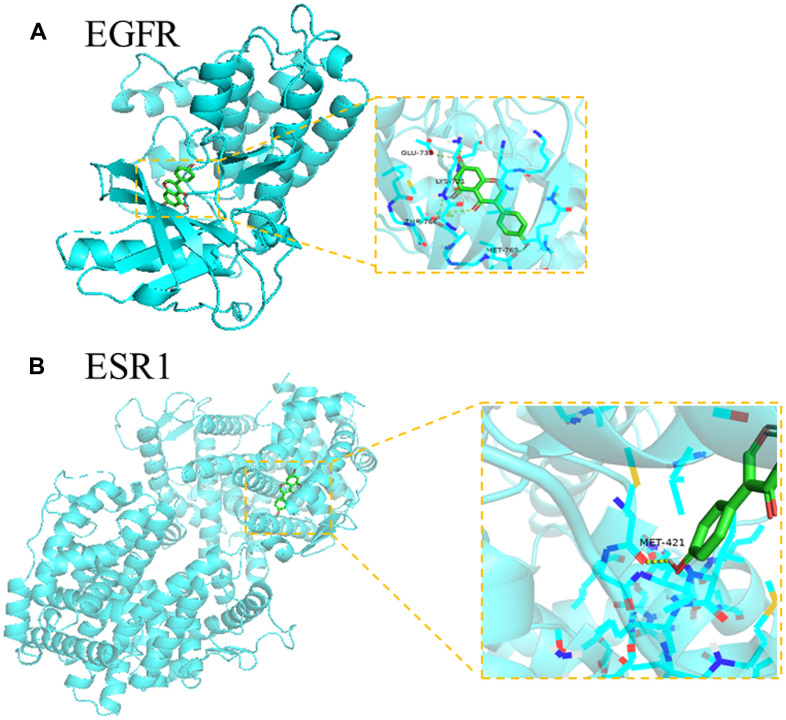
**The molecular docking analysis of genistein and core targets.** (**A**) genistein docking with target EGFR protein (PDB ID:2GS2) in details. (**B**) genistein docking with target ESR1 protein (PDB ID:3OS8) in details.

Following the docking score data, genistein-EGFR and genistein-ESR1 resulted in significant correlation scores. This suggested that EGFR and ESR1 proteins displayed marked affinity for genistein. In EGFR (PDB ID:2GS2), genistein formed hydrogen bonds with the following amino acid residues, MET-769 (2 Å), THR-766 (1.8 Å), LYS-721 (1.9 Å), and GLU-738 (2 Å), and the binding free energy was -7.3 kcal/mol. In ESR1 (PDB ID:3OS8), genistein formed hydrogen bonds with the MET-421 (2.3 Å) amino acid residue, and the binding free energy was -7.7 kcal/mol. Molecular docking data are characterized in Figure 5.

### Clinical validation in human samples

**Figure 6:**
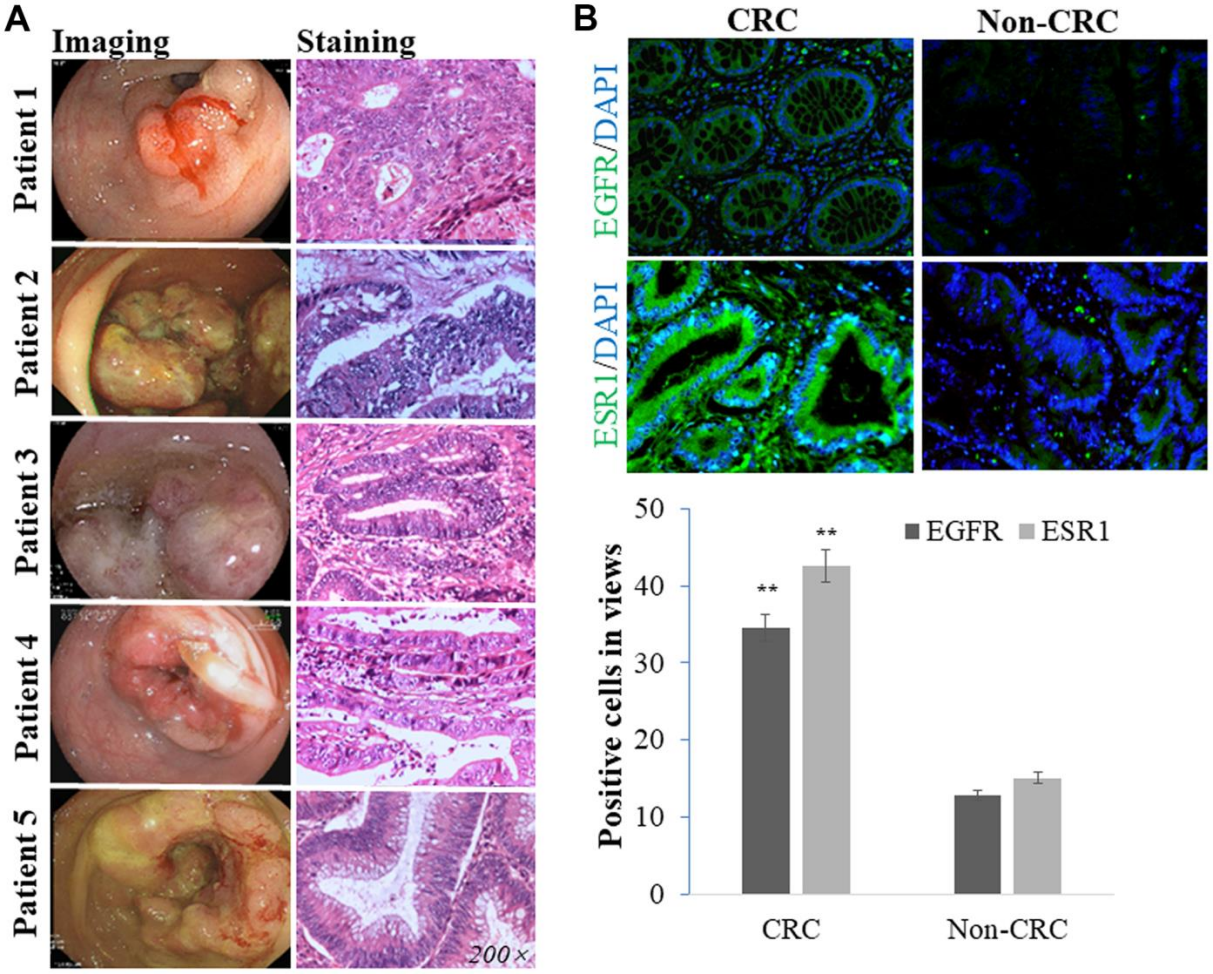
Patients clinically diagnosed with CRC through imaging and HE staining tests (**A**). The intracellular EGFR and ESR1 positive expressions detected in human CRC samples were more than those in non-CRC sections (**B**).

To validate EGFR and ESR1 protein expressions in human CRC and non-CRC samples, we performed immunostaining. The CRC samples were collected from patients with an average age of 55.4 ± 6.1 years. The CRC cases were diagnosed medically using imaging and HE staining tests, as shown in Figure 6A. Immunofluorescence staining results showed that CRC sections resulted in increased endogenous positive cells of EGFR and ESR1 in comparison with those in non-CRC sections (*p* < 0.01, Figure 6B).

### Experimental validation in cell culture

**Figure 7:**
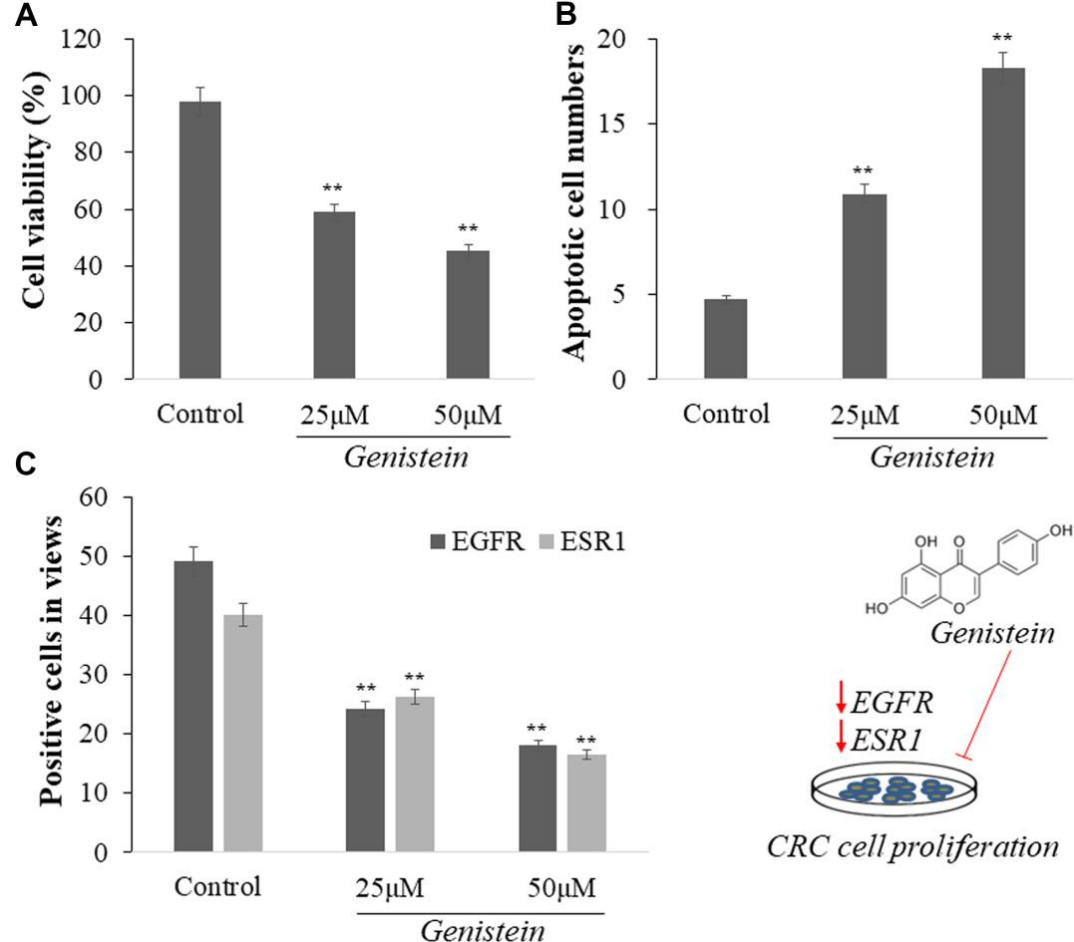
Genistein reduced cell growth and induced apoptosis of CRC cells than those in untreated cells (**A**, **B**). In addition, Genistein-treated CRC cells showed decreased positive cells of EGFR and ESR1 than those in untreated cells (**C**).

A cell line study was performed to validate the molecular docking bioinformatics results. Genistein-dosed treatments showed an increased induction of cell growth suppression in comparison to untreated cells in control group (*p* < 0.01, Figure 7A). Hoechst 33258 staining resulted in elevated apoptosis-positive cells in genistein-treated cells compared to that in untreated cells in control group (*p* < 0.01, Figure 7B). In immunofluorescence staining analysis, genistein-treated groups showed reduced EGFR-positive and ESR1-positive cell quantities in comparison with those in untreated cells in control group (*p* < 0.01, Figure 7C).

## DISCUSSION

Human malignant tumor refers to an obstinate disease characterized by uncontrolled cell growth and division. Autophagy is abnormally modulated in human cancers through facilitating cell proliferation, metastasis and survival. Thus, targeting autophagy may be a promising strategy for the treatment of cancers [40]. Natural anti-cancer compounds characterized by potent treatment action and fewer side effects should be explored in response to clinical challenges, such as drug resistance induced by autophagy and poor prognosis over time [41]. Historically, abundant natural resources are available in China for medicinal and food homology. Traditional Chinese medicine (TCM), with its potential advantages, may pave the way for drug exploration to anage malignancies [42]. Genistein is a Chinese herb-isolated compound with promising properties, including anti-cancer, anti-oxidant, anti-inflammatory, and anti-obesity activities [43]. There is preclinical evidences indicate that genistein exerts anti-CRC properties through suppressing cell differentiation and invasion [44]. The anti-proliferative mechanisms of genistein against CRC may be involved in the modulation of the caspase-3/p38 MAPK pathway for inducing cell apoptosis [45], Akt, and nuclear factor-κB pathway for tumor microenvironmental regulation [46]. However, the molecular mechanisms in genistein against CRC targeting autophagy need to be further explored. In this study, our network pharmacology findings of correlative target genes in genistein and CRC by targeting autophagy suggested that 10 core genes had the highest degree scores, this included microtubule-associated protein tau (MAPT), ESR1, alpha-synuclein (SNCA), matrix metallopeptidase 9 (MMP9), EGFR, cyclin-dependent kinase 1 (CDK1), amyloid precursor protein (APP), CDK5, insulin-like growth factor binding protein 3 (IGFBP3), and glycogen synthase kinase 3beta (GSK3B). Further molecular docking analysis showed that ESR1, and EGFR had the highest affinities for genistein, implying that these core proteins are targeted for autophagy in the treatment of CRC using genistein. Mutations and overexpression of ESR1 have been detected in tumor samples, suggesting potential drug target against human cancers [47]. Methylation of ESR1 has been implicated in the molecular mechanism of endocrinological tolerance in metastatic cancers, including metastatic breast cancer patients [48]. Another study indicated that ESR1 may be a potential pharmacological target for CRC treatment as ESR1 may serve as a tumor-regulated gene via methylation regulation [49]. EGFR is a carcinogenic marker responsible for colorectal oncogenesis and development through regulating EGFR signaling in epithelial cells [50]. EGFR is closely linked to the progression of tumor resistance mechanisms when using anti-cancer medication, accompanied by the marked induction of specific point mutations in EGFR and ALK rearrangements [51]. Abnormally overactivated EGFR has been found in human CRC samples, and EGFR Mutation may be as CRC predictive biomarkers [52]. These reference analyses forementioned imply that genistein may treat CRC by modulating ESR1 and EGFR expressions and activities. Other GO functional data indicated that the top terms/core targets were enriched in the BP, CC, and MF sections. Among these BP-based annotations, heat shock protein binding, protein serine/threonine kinase activity, tau-protein kinase activity, and cyclin-dependent protein serine/threonine kinase activity may be linked to CRC formation and development, and genistein action against CRC. Additionally, other top KEGG pathways were identified using enrichment analysis. The signaling pathways with enriched target genes were detailed in the anti-CRC mechanisms of genistein via targeting of autophagy, including the estrogen signaling pathway. The estrogen signaling pathway is required for many molecular functions, including cell proliferation, gene transcription, and cellular kinase activation [53]. The estrogen signaling pathway is positively associated with CRC cell proliferation via the regulation of key molecular functions [54]. Genistein is a potential anti-CRC candidate that functions with target core genes annotated in the estrogen molecular pathway. Chinese herb-isolated ingredients, including genistein, have a long history of managing human cancers. However, the precise mechanisms and core targets involved remain unclear. In this study, molecular docking analyses validated the feasibility of network pharmacology to identify autophagy targets, and this was confirmed by experimental validation using clinical and cell line samples. In addition, further validated study will be conducted for genistein against CRC targeting autophagy in our future determination.

## CONCLUSIONS

The network pharmacology, molecular docking, and experimental validation results show that genistein may exert the promising anti-CRC effects by targeting autophagy-associated genes and pathways. The results generated from the bioinformatics and biochemical analyses may be useful for future clinical applications of genistein for treating CRC.
